# The systemic inflammatory response and clinicopathological characteristics in patients admitted to hospital with COVID-19 infection: Comparison of 2 consecutive cohorts

**DOI:** 10.1371/journal.pone.0251924

**Published:** 2021-05-27

**Authors:** Donogh Maguire, Conor Richards, Marylynne Woods, Ross Dolan, Jesse Wilson Veitch, Wei M. J. Sim, Olivia E. H. Kemmett, David C. Milton, Sophie L. W. Randall, Ly D. Bui, Nicola Goldmann, Amy Brown, Eilidh Gillen, Allan Cameron, Barry Laird, Dinesh Talwar, Ian M. Godber, John Wadsworth, Anthony Catchpole, Alan Davidson, Donald C. McMillan

**Affiliations:** 1 Emergency Medicine Department, Glasgow Royal Infirmary, Glasgow, United Kingdom; 2 School of Medicine Veterinary and Life Sciences, Wolfson Medical School Building, University of Glasgow, Glasgow, United Kingdom; 3 Academic Unit of Surgery, School of Medicine, University of Glasgow, New Lister Building, Royal Infirmary, Glasgow, United Kingdom; 4 Department of Acute Medicine, Glasgow Royal infirmary, Glasgow, United Kingdom; 5 Institute of Genetics and Molecular Medicine, University of Edinburgh, Edinburgh, United Kingdom; 6 St Columba’s Hospice, Edinburgh, United Kingdom; 7 The Scottish Trace Element and Micronutrient Reference Laboratory, Department of Biochemistry, Royal Infirmary, Glasgow, United Kingdom; 8 Department of Clinical Biochemistry, Queen Elizabeth University Hospital, Govan, United Kingdom; Oregon State University, UNITED STATES

## Abstract

**Background:**

In order to manage the COVID-19 systemic inflammatory response, it is important to identify clinicopathological characteristics across multiple cohorts.

**Methods:**

The aim of the present study was to compare the 4C mortality score, other measures of the systemic inflammatory response and clinicopathological characteristics in two consecutive cohorts of patients on admission with COVID-19. Electronic patient records for 2 consecutive cohorts of patients admitted to two urban teaching hospitals with COVID-19 during two 7-week periods of the COVID-19 pandemic in Glasgow, U.K. (cohort 1: 17/3/2020–1/5/2020) and (cohort 2: 18/5/2020–6/7/2020) were examined for routine clinical, laboratory and clinical outcome data.

**Results:**

Compared with cohort 1, cohort 2 were older (p<0.001), more likely to be female (p<0.05) and have less independent living circumstances (p<0.001). More patients in cohort 2 were PCR positive, CXR negative (both p<0.001) and had low serum albumin concentrations (p<0.001). 30-day mortality was similar between both cohorts (23% and 22%). In cohort 2, age >70 (p<0.05), male gender (p<0.05), COPD (p<0.05), cognitive impairment (p<0.05), frailty (p<0.001), delirium (p = 0.001), CRP>150mg/L (p<0.05), albumin <30 g/L (p<0.01), elevated perioperative Glasgow Prognostic Score (p<0.05), elevated neutrophil-lymphocyte ratio (p<0.001), low haematocrit (p<0.01), elevated PT (p<0.05), sodium <133 mmol/L (p<0.01) elevated urea (p<0.001), creatinine (p<0.001), glucose (p<0.05) and lactate (p<0.001) and the 4C score (p<0.001) were associated with 30-day mortality. In multivariate analysis, greater frailty (CFS>3) (OR 11.3, 95% C.I. 2.3–96.7, p<0.05), low albumin (<30g/L) (OR 2.5, 95% C.I. 1.0–6.2, p<0.05), high NLR (≥3) (OR 2.2, 95% C.I. 1.5–4.5, p<0.05) and the 4C score (OR 2.4, 95% C.I. 1.0–5.6, p<0.05) remained independently associated with 30-day mortality.

**Conclusion:**

In addition to the 4C mortality score, frailty score and a low albumin were strongly independently associated with 30-day mortality in two consecutive cohorts of patients admitted to hospital with COVID-19.

**Trial registration:**

clinicaltrials.gov: NCT04484545.

## Introduction

The number of people worldwide who are known to have been infected with COVID-19 (SARS-CoV-2 infection) increased from 30 million to 84 million in a twelve week period between September 2020 and January 2021, and the number who have died has almost doubled (1 million to 1.8 million) [1]. The severity of this viral disease for an individual is associated with a widespread perturbation of immune, physiological and metabolic parameters [2, 3]. These whole-body changes are characteristic of a systemic inflammatory response to tissue injury. Indeed, measures of this systemic inflammatory response have been shown to have prognostic value [4–6]. In particular, the 4C mortality score was developed in more than 55,000 patients with COVID-19 and measured the systemic inflammatory response using C-reactive protein [6]. Other measures of the systemic inflammatory response such as the neutrophil lymphocyte ratio (NLR) have also been shown to have prognostic value [7]. Moreover, the systemic inflammatory response has been shown to be a useful therapeutic target in patients with COVID-19 [8–10]. However, to date, as there have been variations in the assessment of the systemic inflammatory response, other important factors may remain to be identified. Experience in consecutive cohorts also remains limited.

The aim of the present study was to compare the 4C mortality score, other measures of the systemic inflammatory response and clinicopathological characteristics in two consecutive cohorts of patients on admission with COVID-19.

## Patients and methods

Electronic patient records for patients who were admitted to two large city teaching hospitals (Glasgow Royal Infirmary (GRI) and the Queen Elizabeth University Hospital (QEUH), Glasgow, U.K.), for two consecutive cohorts, cohort 1 (n = 243, 1/4/2020-18/5/2020) [5] and cohort 2 (n = 261, 18/5/2020-6/7/2020) were examined for routine clinical, laboratory and clinical outcome data. These teaching hospitals serve urban populations with a high burden of socio-economic deprivation and offer the full spectrum of adult acute receiving specialties to patients over 16 years old. In line with NHS policy, the NHS Greater Glasgow and Clyde Caldicott guardian approved this study. The study protocol (GN20AE307) was approved by the North West England–Preston research ethics committee (20/NW/0336) and registered with clinicaltrials.gov (NCT04484545). The research ethics committee waived requirement for informed consent and data were gathered from electronic patient records between April 2020 and August 2020.

Details of the recruitment of patients between 1/4/2020-18/5/2020 for cohort 1 have been previously described [5]. In patients admitted to hospital between 18/5/2020-6/7/2020, age, sex, BMI and polymerase chain reaction (PCR) confirmed evidence of COVID-19 infection at time of discharge or death certification were considered minimal criteria for inclusion in cohort 2.

As per routine clinical practice in the Emergency Department (ED) and Acute Assessment Unit (AAU) in both hospitals, patients were scored on the National Early Warning Score (NEWS) at presentation to triage. NEWS is a validated score of severity of physiological derangement that allocates a score (0–3) to six clinical parameters (pulse rate, blood pressure, respiratory rate, oxygen saturations, requirement for supplemental oxygen and level of responsiveness (alert (A), responding to verbal (V), painful (P) stimuli and unresponsive (U) AVPU scale)) [11]. NEWS determines the triage category and level of immediate treatment that is required at the time of presentation, and the interval to re-administering the NEWS scoring tool according to the score achieved (i.e. the severity of physiological derangement). NEWS >4 and >7 are considered to indicate moderately severe and severe physiological derangement respectively.

The 4C Mortality Score is a validated prognostic score that predicts in-hospital mortality among patients with COVID-19 who are admitted to a general hospital setting in the U.K. [6]. It includes eight variables that are readily available at initial hospital assessment: age, sex, number of comorbidities, respiratory rate, peripheral oxygen saturation, level of consciousness, urea level, and C-reactive protein (score range 0–21 points) (see Table 1).

**Table 1:** Final 4C mortality score for in-hospital mortality in patients with COVID-19. Prognostic index derived from penalised logistic regression (LASSO) model [6].

Variable	4C Mortality Score
**Age (years)**	
<50	—
50–59	+2
60–69	+4
70–79	+6
≥80	+7
**Sex**	
Female	—
Male	+1
No of comorbidities*	
0	—
1	+1
≥2	+2
**Respiratory rate (breaths/min)**	
<20	—
20–29	+1
≥30	+2
**Peripheral oxygen saturation on room air (%)**	
≥92	—
<92	+2
**Glasgow coma scale score**	
15	—
<15	+2
**Urea (mmol/L)**	
≤7	—
7–14	+1
>14	+3
**C reactive protein (mg/dL)**	
<50	—
50–99	+1
≥100	+2

* Comorbidities were defined by using Charlson comorbidity index, with the addition of clinician defined obesity

In the present study, age was grouped as less than 40 years, 40–49 years, 50–59 years, 60–69 years, 70–79 years and 80 years and older. Age categories were further simplified to </≥ 70 years. Social deprivation was defined by the Scottish Indices of Multiple Deprivation 2019 based on individual home postcode. Ethnicity was classified as White, Mixed, Asian, Black, or other ethnic group.

Frailty was assessed using the Clinical Frailty Scale (CFS) [12, 13]. The CFS is a validated measure of clinical frailty that has been shown to have prognostic value [13]. The CFS includes items such as comorbidity, cognitive impairment and disability while also incorporating functional interpretation of physical frailty according to level of dependence in living circumstances [12]. In the present study, living circumstances were classified as: independent; living at home with support from family member / paid care assistant or sheltered accommodation; care home; or dependent living in a nursing home.

Admission serum C-reactive protein (CRP), albumin concentrations and differential blood cell counts were categorised using local reference intervals. Neutrophil-lymphocyte ratio (NLR) and the peri-operative Glasgow Prognostic Score (poGPS) were calculated as outlined in Tables 2 and 3 [14–16]. The NLR and poGPS are validated prognostic scoring systems that have been used in a variety of clinical settings. They both utilise two components, neutrophils/ lymphocytes and C-reactive protein/ albumin respectively, that are routinely measured in patients admitted to the general hospital setting. For this study, each scoring system had 3 divisions indicating mild, moderate and severe systemic inflammatory response respectively [17].

**Table 2:** Calculation of the neutrophil lymphocyte ratio (NLR).

Neutrophil Lymphocyte Ratio (NLR):	Ratio	SIRS Severity
**Neutrophil count: lymphocyte count**	<3	Mild
**Neutrophil count: lymphocyte count**	3–5	Moderate
**Neutrophil count: lymphocyte count**	>5	Severe

**Table 3:** Peri-operative Glasgow Prognostic Score (poGPS).

Peri-operative Glasgow Prognostic Score (poGPS)	Score	SIRS Severity
**C-reactive protein ≤ 150mg/l and Albumin ≥25 g/l**	0	Mild
**C-reactive protein > 150mg/l and Albumin ≥25 g/l**	1	Moderate
**C-reactive protein ≤ 150mg/l and Albumin <25 g/l**	1	Moderate
**C-reactive protein > 150mg/l and Albumin <25 g/l**	2	Severe

## Statistical analysis

Demographical and clinicopathological data were presented as categorical variables using recognised clinical thresholds. These variables were analysed using χ^2^ test for linear-by-linear association, or χ^2^ test for 2-by-2 tables.

Associations between demographical, clinicopathological characteristics and mortality were analysed using univariate and a multivariate backward conditional approach. A *p* < 0.05 was applied to inclusion at each step in the multivariate analysis.

A convenience sampling strategy was adopted based on the patients admitted during the study period; therefore, a formal sample size calculation was not performed. Missing data were excluded from analysis on a variable-by-variable basis. Two-tailed *p* values <0.05 were considered statistically significant. Statistical analysis was performed using SPSS software version 27.0. (SPSS Inc., Chicago, IL, USA).

## Results

Details of the recruitment of patients for cohort 1 (n = 243) have been previously described [5]. In cohort 2, of the 356 patients who were confirmed to have COVID-19 infection by PCR test, 278 patients fulfilled the criteria for inclusion with age, sex, BMI. Seventeen patients were re-admitted and these were excluded from the analysis at second admission leaving 261 patients to be included in the analysis.

Comparison of the demographical and clinicopathological characteristics of the two cohorts are shown in Table 4. Compared with cohort 1, cohort 2 were older (p<0.001), more likely to be female (p<0.05) and have less independent living circumstances (p<0.001). With reference to previous medical history, compared with cohort 1, cohort 2 had hypertension and heart failure (both p<0.05), had chronic renal failure (p<0.001), had cognitive impairment and previous delirium (both p<0.01), were less frail (p<0.001) and had less asthma (p<0.01). With reference to diagnostic criteria, compared with cohort 1, cohort 2 were more likely to be PCR positive and CXR negative (both p<0.001). With reference to laboratory results, compared with cohort 1, cohort 2 had low albumin (p<0.001), low haemoglobin (p<0.001), low haematocrit (p<0.05), lower MCV (0.05), abnormal sodium (p<0.01), elevated creatinine (p<0.01), elevated alkaline phosphatase (p<0.001). 30-day mortality was similar between the cohorts (23% and 22%).

**Table 4:** Comparison of the demographical and clinicopathological characteristics of 2 consecutive cohorts patients admitted to hospital with COVID-19.

	Cohort 1 (n = 243)		Cohort 2 (n = 261)		p-value
	n	%	n	%	
30-days post admission (alive /dead)	188/55	77/23	203/58	78/22	0.912
Age (</≥70 years)	157/86	65/35	89/172	34/66	<0.001
Sex (male/female)	133/110	55/45	119/142	46/54	0.041
BMI (<20; ≥20–29; ≥30 kg/m^2^)	18/119/106	7/44/49	42/139/80	16/53/31	0.475
SIMD (1 (most)– 6 (least) deprived)	124/35/25/26/22/11	51/14/10/11/9/5	98/49/27/29/32/20	38/19/11/11/13/8	0.011
Ethnicity (1–5)	209/0/7/2/5	93/0/4/1/2	239/1/20/1/0	91/0.4/8/0.4/0	0.818
Living circumstances (0–5)	206/16/15/4/2	84/7/6/2/1	178/18/24/41/0	68/7/9/16	<0.001
**Past Medical History**					
Hypertension (y/n)	96/147 (40/60)	39/61	129/132	49/51	0.025
Heart failure (y/n)	23/220	10/90	42/219	16/84	0.027
T1DM (y/n)	2/241	1/99	2/259	1/99	0.943
T2DM (y/n)	58/185	24/76	61/199	24/76	0.915
Chronic renal failure (y/n)	29/214	12/88	62/199	24/76	0.001
Cognitive impairment (y/n)	31/212	13/87	85/176	33/67	<0.001
Previous delirium (y/n)	16/225	7/93	41/219	16/84	0.001
Clinical frailty score (≤/> 3)	134/109	55/45	75/185	71/29	<0.001
COPD (y/n)	41/202	17/83	47/214	18/82	0.738
Smoker (never/ex/active)	82/70/20	48/42/10	24/27/1	56/38/6	0.428
Alcohol excess (y/n)	33/210	14/86	30/231	12/88	0.480
Liver disease (y/n)	20/223	8/92	12/249	5/95	0.095
Hep C (never/previous/active)	237/3/2	98/1/1	260/0/1	99/0.5/0	0.166
Active cancer (y/n)	11/232	5/95	16/245	6/94	0.425
Asthma (y/n)	47/196	20/80	24/237	9/91	0.001
**Diagnostic criteria**					
PCR positive/Clinical Dx. /Radiological Dx	120/8/115	50/3/47	261/0/0	100/0/0	<0.001
PCR negative/indeterminate/positive	49/54/136	20/22/58	0/24/237	0/8/92	<0.001
CXR negative/positive	101/138	42/58	148/98	60/40	<0.001
**Physiology at presentation**					
NEWS (≤ / > 4)	92/149	38/62	126/123	51/49	0.006
Delirium (y/n)	26/211	11/89	54/179	23/77	<0.001
**Laboratory results at presentation**					
CRP (<150 / ≥150 mg/L)	182/60	75/25	211/49	81/19	0.107
Albumin (≥35/<35 g/L)	104/135	43/57	75/179	30/70	<0.001
poGPS (0/1/2)	176/55/7	72/23/3	192/53/8	76/21/3	0.711
WCC (< 4.5 / ≥4.5 - ≤11.0 / >11.0 x 10^9^/L)	36/165/41	68/15/17	44/160/53	62/17/21	0.176
Neutrophils (< / ≥ 7.5 x 10^9^/L)	183/59	76/24	185/71	72/28	0.395
Lymphocytes (≥ / < 1.5 x 10^9^/L)	64/176	26/74	62/162	24/76	0.415
NLR (<3/ 3–5 /≥ 5)	52/58/130	22/24/54	60/63/133	23/25/52	0.587
Hb (≥/<12.0 g/dL)	195/46	81/19	163/93	64/36	<0.001
Hct (male ≥/< 0.40) (female ≥/< 0.37) L/L	169/73	70/30	153/104	60/40	0.016
MCV (<80/ ≥80 - <99/≤ 99 fl)	6/218/16	3/91/7	5/207/45	2/80/18	0.011
Platelets (< 150/ ≥150 - <400/>400x10^9^)	16/188/9	78/18/4	33/197/25	13/77/10	0.103
Sodium (<133/≥133- ≤146/>146 mmol/L)	28/210/5	12/86/4	41/204/16	16/78/6	0.006
Potassium (<3.5/≥3.5- ≤5.5/>5.5 mmol/L)	22/192/2	10/89/1	29/206/7	12/85/3	0.135
Mg (≥/< 0.75 mmol/L)	41/88	69/31	38/30	56/44	0.063
Urea (≤/> 7.5 mmol/L)	162/81	67/33	150/111	58/42	0.034
Creatinine (≤/>130 umol/L)	218/25	90/10	208/53	80/20	0.002
AST (≤/ > 40 IU)	131/78	63/37	153/74	67/33	0.302
ALT (≤/ > 56 IU)	203/35	85/15	211/43	83/17	0.500
AST: ALT </≥ 2	180/29	86/14	183/43	81/19	0.149
ALP (≤/ > 130 IU)	221/18	93/7	198/56	78/22	<0.001
Bilirubin (≤/ > 17 mmol/L)	213/26	89/11	217/37	85/15	0.221
Glucose (≤/ > 7 mmol/L)	123/81	60/40	124/58	68/32	0.110
Lactate (</ ≥ 2 mmol/L)	60/35	64/36	60/45	57/43	0.299
HCO_3_ (≥ / < 22 mmol/L)	46/12	80/20	66/33	67/33	0.092
PT (≤ / > 13 seconds)	125/70	64/36	94/55	63/37	0.846
APPT (≤ / > 38 seconds)	177/12	94/6	126/22	85/15	0.010
Alive/Dead at 30-days	188/55	77/23	203/58	78/22	0.912
**Level of care (ward/HDU/ITU)**					
Initial level of care	199/15/8	89/7/4	236/13/6	93/5/2	0.261
Max level of care	159/38/25	72/17/11	228/13/14	89/5/6	<0.001

poGPS: Peri-operative Glasgow Prognostic Score; NLR: neutrophil lymphocyte ratio

The relationship between demographic and clinicopathological characteristics and 30-day mortality in cohort 2 is shown in Table 5. In cohort 2, age ≥70 (p<0.05), male gender (p<0.05), COPD (p<0.05), cognitive impairment (p<0.05), frailty (p<0.001), delirium (p = 0.001), CRP≥150mg/L (p<0.05), albumin <30 g/L (p<0.01), elevated perioperative Glasgow Prognostic Score (p<0.05), elevated neutrophil-lymphocyte ratio (p<0.001), low haematocrit (p<0.01), elevated PT (p<0.05), sodium <133 mmol/L (p<0.01) elevated urea (p<0.001), creatinine (p<0.001), glucose (p<0.05) and lactate (p<0.001) and the 4C score (p<0.001) were associated with 30-day mortality.

**Table 5:** Univariate analysis of demographical, clinicopathological characteristics and 30-day mortality in the second cohort of patients admitted with confirmed COVID-19 (n = 261).

	Alive (n = 203)	Dead (n = 58)	p-value
Age (</≥70 years)	77/126	12/46	0.015
Sex (male/female)	85/118	34/24	0.024
BMI (<20; ≥20–29; ≥30 kg/m^2^)	27/104/72	15/35/8	0.756
SIMD (1 (most)– 6 (least) deprived)	80/36/21/18/29/15	18/13/6/11/3/5	0.777
Ethnicity (1–5)	185/1/16/1/0	54/0/4/0/0	0.639
Living circumstances (0–4)	146/14/16/27	32/4/8/14	0.010
**Past Medical History**			
Hypertension (y/n)	96/107	33/25	0.198
Heart failure (y/n)	31/172	11/47	0.500
T1DM (y/n)	1/202	1/57	0.344
T2DM (y/n)	43/159	18/40	0.123
Chronic renal failure (y/n)	43/160	19/39	0.068
Cognitive impairment (y/n)	59/144	26/32	0.024
Previous delirium (y/n)	31/171	10/48	0.728
Clinical frailty score (≤/> 3)	130/72	55/3	<0.001
COPD (y/n)	32/171	15/43	0.078
Smoker (never/ex/active)	119/71/13	27/29/2	0.312
Alcohol excess (y/n)	25/178	5/53	0.437
Liver disease (y/n)	9/194	3/55	0.813
Hep C (never/previous/active)	203/0/0	57/0/1	0.061
Active cancer (y/n)	11/192	5/53	0.371
Asthma (y/n)	22/181	2/56	0.086
**Diagnostic radiology**			
CXR negative/positive	119/72	26/29	0.202
**Physiology at presentation**			
NEWS (≤ / > 4)	103/90	23/33	0.106
Delirium (y/n)	149/33	30/21	0.001
**Laboratory results at presentation**			
CRP (< / ≥150 mg/L)	170/32	41/17	0.021
Albumin (≥/<30 g/L)	52/146	30/26	0.004
poGPS (0/1/2)	156/35/6	36/18/2	0.047
WCC (< 4.5 / ≥4.5 - ≤11.0 / >11.0 x 10^9^/L)	38/127/34	6/33/19	0.062
Neutrophils (< / ≥ 7.5 x 10^9^/L)	155/43	30/28	<0.001
Lymphocytes (≥ / < 1.5 x 10^9^/L)	55/142	7/50	0.016
NLR (<3/ 3–5 /≥ 5)	56/58/84	4/5/49	<0.001
Hb (≥/<12.0 g/dL)	125/73	38/20	0.740
Hct (male ≥/< 0.40) (female ≥/< 0.37) L/L	122/77	31/27	0.284
MCV (<80/ ≥80 - <99/≤ 99 fl)	5/162/32	0/45/13	0.848
Platelets (< 150/ ≥150 x10^9^)	22/175	11/47	0.121
Sodium (<133/≥133- ≤146/>146 mmol/L)	32/164/7	9/40/9	0.005
Potassium (<3.5/≥3.5- ≤5.5/>5.5 mmol/L)	44/160/4	7/46/3	0.304
Mg (≥/< 0.75 mmol/L)	71/35	25/8	0.343
Urea (≤/> 7.5 mmol/L)	133/70	17/41	<0.001
Creatinine (≤/>130 umol/L)	177/26	31/27	<0.001
AST (≤/ > 40 IU)	121/54	32/20	0.305
ALT (≤/ > 56 IU)	160/38	51/5	0.071
AST: ALT </≥ 2	145/29	38/14	0.099
ALP (≤/ > 130 IU)	158/40	40/16	0.183
Bilirubin (≤/ > 17 mmol/L)	173/25	44/12	0.100
Glucose (≤/ > 7 mmol/L)	101/37	23/21	0.010
Lactate (</ ≥ 2 mmol/L)	50/23	10/22	<0.001
HCO_3_ (≥ / < 22 mmol/L)	52/20	14/13	0.057
PT (≤ / > 13 seconds)	78/36	16/19	0.015
APPT (≤ / > 38 seconds)	95/17	31/5	0.850
4C score (0 - <4 / ≥4 - <9 / ≥9 - <15 / ≥15)	26/65/84/8	1/7/32/13	<0.001

In the combined cohorts (n = 504), there was no association between serum sodium concentrations and use of medications such as proton pump inhibitors (p = 0.119), angiotensin converting enzyme inhibitors (ACEi’s) (p = 0.608) or diuretics (p = 0.675).

To determine which admission parameters were independently associated with 30-day mortality, those factors identified in Table 5 as significant and not in the 4C mortality score were also entered into a binary logistic regression analysis (Table 6). In this multivariate analysis, only greater frailty (CFS>3) (OR 11.3, 95% C.I. 2.3–96.7, p<0.05), low albumin (<30g/L) (OR 2.5, 95% C.I. 1.0–6.2, p<0.05) and high NLR (≥3) (OR 2.2, 95% C.I. 1.5–4.5, p<0.05) and the 4C score (OR 2.4, 95% C.I. 1.0–5.6, p<0.05) remained independently associated with 30-day mortality.

**Table 6:** Binary logistic regression analysis of demographical, clinicopathological characteristics and 30-day mortality in the second cohort of patients admitted with confirmed COVID-19 (n = 203).

	Alive (n = 125)	Dead (n = 38)	p-value	).R.	95% CI	p-value
Clinical frailty score (≤/> 3)	79/63	12/49	<0.001	11.3	1.3–96.7	0.027
Albumin (≥/<30 g/L)	119/23	39/22	0.002	2.5	1.0–6.2	0.045
NLR (<3/≥3 - <5/≥ 5)	28/37/77	3/8/50	<0.001	2.2	1.0–4.5	0.040
Hct (male ≥/< 0.40) (female ≥/< 0.37) L/L	106/36	35/26	0.015			0.554
Sodium (<133/≥133 - ≤146/>146 mmol/L)	15/123/4	12/41/8	<0.001			0.321
Glucose (≤/ > 7 mmol/L)	80/49	24/30	0.029			0.076
4C score (0 - <4 / ≥4 - <9 / ≥9 - <15 / ≥15)	19/56/50/6	0/6/38/14	<0.001	2.4	1.0–5.6	0.049

Hct: Haematocrit

## Discussion

The results of the present study show that, in 2 consecutive cohorts, there was a variation in the admission demographic and clinicopathological characteristics. In particular, the latter cohort were older, had more cardiovascular and renal disease and greater derangement of their laboratory data. Despite this, 30-day mortality was similar between the cohorts. In both cohorts the 4C score, which incorporates age, sex, comorbidity, respiratory, renal and brain function and a measure of the activation of the systemic inflammatory response, had prognostic value. In addition, when compared directly with the 4C mortality score a number of other factors had independent prognostic value, in particular clinical frailty and a low albumin. Taken together, the present results would suggest that the relationship between clinicopathological factors and short-term mortality in patients with COVID-19 may vary with time. Also, that there are other important factors in short term mortality of patients with COVID-19, not captured by the 4C mortality score and such factors may improve the prognostic value of the 4C score. Therefore, there is a need for further work to determine independent prognostic value of clinicopathological factors in patients presenting with COVID-19.

Dysnatraemia (both hyponatremia and hypernatremia) are known to be associated with mortality and sepsis, but only hyponatremia is usually associated with intensive therapy. Admittance hyponatraemia is recognised to be associated with increased mortality. Furthermore, hyponatremia has been reported to be a risk factor for infection, specifically for Staphylococcus aureus bacteremia [18]. In the present study, the majority of patients had haematocrit concentrations within normal parameters and there was no significant association between haematocrit and serum sodium concentrations, however data relating to the presence of bacterial supra-infections was not gathered in the present study. Also, in relation to dysnatraemia, it has been reported that pro-inflammatory cytokines particularly IL-1b and IL-6 can stimulate hypothalamic Arginine Vasopressin secretion and that circulating IL-6 concentrations are inversely associated with Na concentrations in patients with COVID-19 sepsis [19]. In the present study IL-6 concentrations were not measured and data relating to treatment with specific anti-IL-6 agents was not collected. However, data was collected on proton pump inhibitors (PPi’s), angiotensin converting enzyme inhibitors (ACEi’s), diuretics, NSAIDs, Ca^2+^ blockers, immuno-suppressants (including steroids) prior to presentation and in the combined cohorts (n = 504), there was no association between serum sodium concentrations and use of medications such as PPi’s, ACEi’s or diuretics.

In the present study admittance hypoalbuminaemia was a predictive factor for COVID-19 outcomes. The results of the present study are also consistent with a meta-analysis of sixty-seven studies in 19,760 COVID-19 patients (6,141 with severe disease or poor outcome) reported by Paliogiannis and co-workers that lower serum albumin concentrations were significantly associated with disease severity and adverse outcomes in COVID-19 patients [20]. As such, they suggest that serum albumin concentrations might assist with early risk stratification and selection of appropriate care pathways for patients with COVID-19 infection. Indeed, it is recognised that as part of the systemic inflammatory response there is escape of serum albumin into the interstitial space due to increased capillary permeability resulting in lower circulating albumin concentrations. Therefore, it may be concluded that hypoalbuminaemia is a useful therapeutic target and that albumin supplementation would be an effective therapeutic strategy. However, there are parallels with patients who are critically-ill secondary to other infections and albumin supplementation has not been associated with improved clinical outcomes [21].

In the present study, the finding that CFS>3 was independently associated with eleven times higher 30-day mortality in patients admitted to hospital with COVID-19 SIRS is consistent with a recently published meta-analysis by the Geriatric Medicine Collaborative. In their meta-analysis of data from 5,711 patients admitted to hospital with COVID-19 SIRS, the Geriatric Medicine Collaborative report that very high frailty scores (CFS>8) (‘Dependent’ and ‘Bed-bound’) was independently associated with 30-day mortality when compared to non-frail patients (CFS<3) (CFS 8 v^s^ 1–3: HR 3.03, CI 2.29–4.00) [22]. The findings of the present study may offer a valuable insight in this context as it includes patients who are classified in the moderate frailty range (CFS4-6).

The basis of the strong prognostic value of the clinical frailty scale and low albumin, independent of the 4C mortality score in these patients, is not clear. However, COVID-19 patients with a clinical frailty score >3 may be considered vulnerable and frail and this entity (the ability to care for themselves and its relationship with mortality) may not be captured directly by the 4C score. In particular, the present results may indicate that having COVID-19 induced cytokine storm in a frail patient is a life threatening event [23]. Similarly, with reference to a low serum albumin concentration, a cytokine storm would increase the likelihood of mortality. In the case of a low albumin it is clear that this may reflect both an ongoing systemic inflammatory response and also poor nutritional status [24]. It may be that the strong prognostic value of frailty also reflects poor nutritional status since a systemic inflammatory response occurring against a background of low metabolic reserves is likely to lead to cellular and organ dysfunction. If this was the case, then it might be expected that frail and hypoalbuminaemic patients would benefit most from treatment with anti-inflammatory agents and nutritional supplementation. Therefore, it may be important to also consider nutritional risk in patients with COVID-19. Irrespective, it would be important to consider frailty and a low albumin in the assessment of patients with COVID-19 [25].

To date, in patients with COVID-19, there has been a great deal of focus on the virus itself. However, it is clear from the prognostic value of host physiology and the host systemic inflammatory response in the 4C work [6] and in the efficacy of dexamethasone treatment [13] that host factors are of considerable importance in outcome of patients with COVID-19. From the present results it is also clear that frailty and nutritional status are important characteristics to be taken into the “staging” of patients presenting with COVID-19.

### Limitations

The present study has a number of limitations. The sample size is relatively small and therefore subject to limitations such as sample bias. However, the clinicopathological data collected was comprehensive across two cohorts, included factors validated in large cohorts of patients with COVID-19 and therefore allowed direct comparison of these factors.

In the present study, two cohorts of patients with COVID-19 infection were studied, reflecting the rapidly evolving clinical reality of presentation in the early stages of the pandemic. For example, PCR testing was not as readily available in the first cohort and clinicians were required to make COVID-19 diagnosis based on WHO and Scottish Department of Health criteria. Therefore, it is perhaps not surprising that there were a number of differences between the cohorts. However, this is likely to be an issue in many of the reports of clinicopathological factors and clinical outcomes in patients with COVID-19.

### Conclusion

In the two consecutive cohorts there were variations in a number of clinicopathological characteristics despite similar mortality. In these two cohorts, in addition to the 4C mortality score, NLR≥3, clinical frailty score >3 and low serum albumin concentration (<30 g/L) were independently associated with 30-day mortality in patients admitted to hospital with COVID-19 infection.
